# A spectacular new species of seadragon (Syngnathidae)

**DOI:** 10.1098/rsos.140458

**Published:** 2015-02-18

**Authors:** Josefin Stiller, Nerida G. Wilson, Greg W. Rouse

**Affiliations:** 1 Scripps Institution of Oceanography, UCSD, 9500 Gilman Drive, La Jolla, CA 92093-0202, USA; 2 Western Australian Museum, 69 Kew St., Welshpool 6106, Perth, Western Australia, Australia

**Keywords:** biodiversity, Syngnathidae, new species, seadragon

## Abstract

The exploration of Earth's biodiversity is an exciting and ongoing endeavour. Here, we report a new species of seadragon from Western Australia with substantial morphological and genetic differences to the only two other known species. We describe it as *Phyllopteryx dewysea* n. sp. Although the leafy seadragon (*Phycodurus eques*) and the common seadragon (*Phyllopteryx taeniolatus*) occur along Australia's southern coast, generally among relatively shallow macroalgal reefs, the new species was found more offshore in slightly deeper waters. The holotype was trawled east of the remote Recherche Archipelago in 51 m; additional specimens extend the distribution west to Perth in 72 m. Molecular sequence data show clear divergence from the other seadragons (7.4–13.1% uncorrected divergence in mitochondrial DNA) and support a placement as the sister-species to the common seadragon. Radiographs and micro-computed tomography were used on the holotype of the new species and revealed unique features, in addition to its unusual red coloration. The discovery provides a spectacular example of the surprises still hidden in our oceans, even in relatively shallow waters.

## 2. Introduction

The fraction of undescribed biodiversity in the ocean is arguably high [1,2], and new fish species are likely to be described, especially from the deeper continental slopes [3]. Here, we describe a remarkable example, a new species of seadragon. *Phyllopteryx dewysea* n. sp. is only the third known species of seadragon, and the first to be discovered in 150 years, possibly because it lives in slightly deeper waters than its relatives.

**Figure 1.**
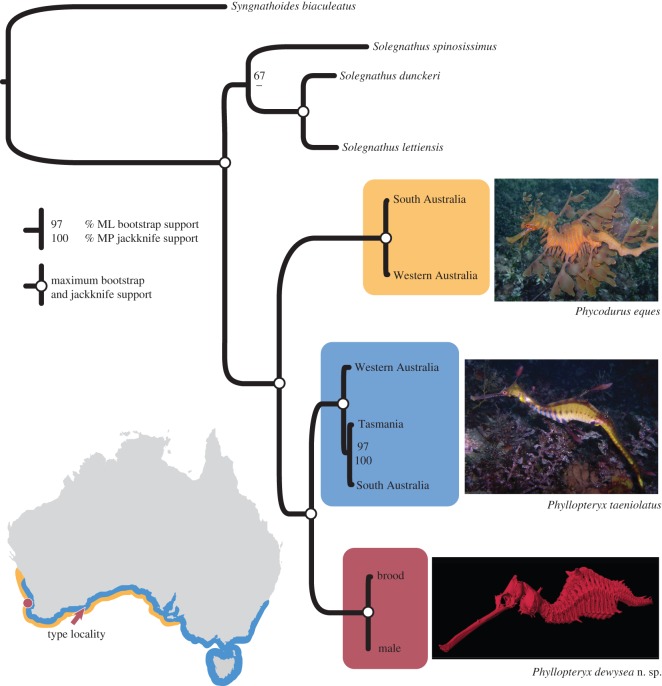
Distribution and phylogenetic relationships of the three seadragon species. The range of the leafy seadragon is shown in gold, the common seadragon in blue and the type locality of the new seadragon *Phyllopteryx dewysea* n. sp. is indicated by the red arrow. The red circle represents the collection sites of the three paratypes. The maximum-likelihood tree is based on the partitioned dataset (4416 bp). Numbers next to the nodes indicate bootstrap (top) and jackknife (bottom) values; full support is indicated by white circles.

Seadragons (Syngnathidae) are fish of mesmerizing beauty (figure 1), with the leafy seadragon (*Phycodurus eques*) having more elaborate appendages than the colourful common seadragon (*Phyllopteryx taeniolatus*). Their ornamentation helps camouflage them among seagrasses and kelp in the shallow coastal waters of southern Australia [4]. The range of the common seadragon spans the entire southern coast from Western Australia to New South Wales and Tasmania, whereas the leafy seadragon has a more restricted distribution from Western Australia to South Australia (map in figure 1). The two known species of seadragons fascinate aquarium visitors around the world, draw many SCUBA divers into the water and are charismatic symbols for conservation of marine species [5,6].

On a biodiversity survey (part of the Marine Futures project 2007; http://www.marinefutures.fnas.uwa.edu.au/), a male seadragon carrying a brood of offspring was trawled off the coast of Western Australia. The specimen was accessioned at the Western Australian Museum (WAM P33223.002) as a common seadragon, and its uniqueness only subsequently recognized following DNA sequencing. Three additional specimens were located in scientific collections. The holotype of the new seadragon shows a bright red coloration and with transverse body-markings similar to leafy seadragons. However, the body shape more closely resembles the common seadragon. Here, we examine the anatomy and evolutionary history of the new seadragon to determine its systematic placement and formally describe it.

## 3. Material and methods

### 3.1 Sampling

Several diagnostic features of the skeleton allowed us to identify additional specimens of the new species that had been catalogued in scientific collections as common seadragons, even when they had lost colour and were unsuitable for genetic analysis. As the holotype was trawled from an unusual depth, we requested and examined specimens from depths of more than 50 m. Most museum specimens, however, do not hold explicit depth records, therefore we also asked for information on individuals collected on vessels, as those are more likely to have been collected offshore. A total of 15 trawled specimens from New South Wales, Tasmania, South Australia and Western Australia (see the electronic supplementary material for details) were located. Of these, two specimens (CSIRO Australian National Fish Collection; ANFC C2269 and C2270) collected near Perth, Western Australia, were trawled from a depth of 72 m and are here designated as paratypes of the new species based on their anatomical similarity to the holotype. The other trawled specimens were common seadragons, unfortunately mostly without depth records, which makes it difficult to infer a depth range for this species. The deepest record for a common seadragon was from 27 to 33 m (WAM P.20854; see the electronic supplementary material for details). We visually inspected 158 lots of common seadragons held at the Western Australian Museum, mostly from the southwestern coast of Australia, and found an additional paratype from Perth without a depth record as it was beachwashed. No specimens of the new species are housed at the South Australian Museum (Ralph Foster 2014, personal communication), which holds 60 lots of common seadragons mainly from South Australia and Victoria.

### 3.2 Molecular sampling

A piece of the tail of the holotype (preserved in ethanol) was used for DNA extraction as the dermal appendages were missing in this species; the specimen was a male carrying its brood, and we also obtained DNA sequences for one of its brood of embryos in order to obtain the mitochondrial haplotype of the mother. A DNA extraction protocol for formalin-preserved tissues [7] was unsuccessful for two of the paratypes (ANFC C2269 and C2270) and not attempted for the third paratype (WAM P660) because of its advanced age (collected 1919). For comparison with the other two seadragon species, we included genetic samples of wild-caught common seadragons and leafy seadragons from across their respective ranges (table 1). A small tissue clip was taken from the ventral dermal appendages of live animals that were subsequently released. This non-lethal sampling was considered less destructive than fin-clipping as the dermal appendages do not seem to have a locomotory function and are often damaged in otherwise healthy seadragons. We included three specimens of the common seadragon from Western Australia (Shelley Cove, Dunsborough, 17 December 2005), South Australia (Encounter Bay, 8 April 2006) and Tasmania (Blackmans Bay, Hobart, 16 December 2012). For the leafy seadragon, we included two specimens, one from South Australia (Encounter Bay, 3 February 2006), the other from Western Australia (Bremer Bay, 11 December 2005). See the ethics statement for permit details.

**Table 1.** Collection details and GenBank accession numbers. WA, Western Australia; SA, South Australia; TAS, Tasmania.

species	locality	12S	16S	ND4	control region	S7	Tmo-4c4	aldolase
*Phyllopteryx dewysea*n. sp.	WA, east of Middle Island (male, brood)	KM201546	KM201553	KM201563	KM201574	KM201593	KM201604	KM201583
		KM201547	KM201554	KM201564	KM201575	KM201594	KM201603	KM201584
*Phycodurus eques*	SA, Encounter Bay	GU182919	GU182927	KM201558	KM201569	KM201588	KM201598	KM201578
	WA, Bremer Bay	GU182918	GU182926	KM201559	KM201570	KM201589	KM201599	KM201579
*Phyllopteryx taeniolatus*	SA, Encounter Bay	KM201544	KM201551	KM201561	KM201571	KM201590	KM201602	KM201580
	TAS, Hobart	KM201545	KM201552	KM201562	KM201572	KM201591	KM201601	KM201581
	WA, Dunsborough, Shelley Cove	GU182920	GU182928	KM201560	KM201573	KM201592	KM201600	KM201582
*Solegnathus dunckeri*	—	JF273446	GU182924	—	—	—	—	—
*Solegnathus lettiensis*	WA, northwest of Rottnest Island	KM201550	KM201557	KM201567	KM201576	KM201586	KM201596	—
*Solegnathus spinosissimus*	New Zealand, Southland, Milford Sound	KM201549	KM201556	KM201566	—	KM201587	KM201597	KM201577
*Syngnathoides biaculeatus*	Aquarium specimen	KM201548	KM201555	KM201565	KM201568	KM201585	KM201595	—

Outgroup samples included three members of Solegnathinae, a group which was identified as the sister-group to the seadragons [8–10]. We included sequences from three species of spiny pipehorses, namely *Solegnathus spinosissimus* (Auckland Museum Marine Collections MA133942, New Zealand, Southland, Milford Sound, total length (TL) 1900 mm, May 2006), *Solegnathus dunckeri* (from GenBank, table 1), and *Solegnathus lettiensis*(ANFC H6340-09/GT192, Western Australia, northwest of Rottnest Island, RV Naturaliste, 100 m depth, 7 April 2006, supplied as *Solegnathus guentheri*). We also included *Syngnathoides biaculeatus* (Syngnathoidinae; gift from the Birch Aquarium at Scripps, 28 May 2013), which was shown to be closely related to seadragons and Solegnathinae [8–10].

### 3.3 DNA extraction and PCR amplification

DNA was isolated with the Qiagen DNeasy blood and tissue kit (Qiagen Sciences, Germantown, MD). Seven marker regions (four mitochondrial, three nuclear) were amplified in a reaction volume of 12.5 μl with the following primer pairs:

— *12S*: L1091 and H1478 [11], modified following reference [9], amplified 346 bp of the 12S ribosomal RNA gene, using the thermal profile of 95°C for 15 min, 35 cycles of 95°C for 40 s, 50°C for 40 s, 68°C for 50 s, and a final extension at 68°C for 5 min.
— *16S*: L2510 and H3058 [12], which amplify 522 bp of the 16S ribosomal RNA gene, with the same thermal profile as 12S.
— *ND4*: L11424-ND4 [13] and H12293-Leu [14], amplified an 838 bp fragment of the NADH dehydrogenase subunit four, including transfer RNA histidine and serine, with a thermal profile of 94°C for 9 min, 34 cycles of 94°C for 45 s, 52°C for 45 s, 72°C for 60 s, and a final extension at 72°C for 6 min.
— *Control region*: L15995 L-PRO [15] and H693.5 12S5r.5 [16], amplified a 1036 bp fragment of the mitochondrial control region, including fragments of the 12S ribosomal RNA gene. The fragment has a repetitive region in the middle of the fragment that impaired the sequencing reaction. To obtain the full-length fragment, we developed an additional primer set for sequencing the flanking regions of the mitochondrial control region (CR-FlanksF 5^′^-CCCCCACCCCCTTTAAAGAC-3^′^ and CR-FlanksR 5^′^-CGAGTCGTATGTGTCCCACC-3^′^). All fragments were amplified via a thermal profile of 94°C for 3 min, 40 cycles of 94°C for 60 s, 50°C for 30 s, 72°C for 60 s, and a final extension at 72°C for 5 min.
— *S7*: S7RPEX1F and S7RPEX2R [17] amplified up to 674 bp of the intron 1 and exon 2 of the nuclear S7 ribosomal protein gene with the thermal profile specified in reference [18].
— *Aldolase*: we used forward and reverse primers as specified in reference [19], amplifying a 382 bp long fragment of a nuclear aldolase-like gene containing both coding and non-coding regions with a thermal profile as outlined in reference [19].
— *Tmo-4c4*: Tmo-4c4F and Tmo-4c4R [20] amplified a 559 bp long coding fragment of the nuclear Tmo-4c4 gene with the thermal profile specified in reference [18].

PCR products were purified with Exo-SAP-IT (USB Affymetrix) and sequencing was completed with the corresponding PCR primers (Eurofins MWG Operon, Inc.). Overlapping fragments were edited and then merged into consensus sequences in Geneious v. 6.0.3 (Biomatters Ltd.). All sequences were deposited on GenBank (table 1).

Sequences for each marker were aligned on the MAFFT v. 7 server [21,22]. Most markers aligned without gaps, but gaps were inserted into the control region fragment resulting in an aligned length of 1084 bp, and S7-like protein aligned to a length of 685 bp. Alignments were deposited on TreeBASE (S15971).

### 3.4 Distance-based analysis

Uncorrected and model-corrected genetic distances were calculated in PAUP* v. 4b10 [23]. The appropriate model selected by jModelTest v. 2.1.4 [24] employing the Akaike information criterion was GTR + G for the mitochondrial dataset (control region, 16S, 12S, ND4) with a gamma parameter of 0.283. For S7 and aldolase, the chosen model was HKY, and K80 + I for Tmo-4c4 with a proportion of invariant sites of 0.723.

### 3.5 Phylogenetic analysis

To assess the phylogenetic affinities of the seadragons, we conducted maximum-parsimony (MP) and maximum-likelihood (ML) analyses. To check for data congruence, we analysed each nuclear marker individually, while we treated the mitochondrial markers as a single locus (2790 bp), and finally concatenated the datasets (4416 bp) in Mesquite v. 2.75 [25].

Maximum-parsimony analyses were conducted in PAUP*, with unordered and equally weighted characters, heuristic searches with 1000 random addition-sequence replicates and tree bisection and reconnection (TBR) branch swapping and with zero-length branches collapsed. To assess clade confidence, parsimony jackknifing was conducted with 1000 jackknife replicates, each with 100 random addition replicates, 37% character deletion, heuristic search option and TBR branch swapping.

Maximum-likelihood analyses were conducted in raxmlGUI v. 0.93 [26,27] employing the GTR + G model for each of the partitions. The dataset was partitioned for the different loci, for introns and exons of aldolase and S7, and additionally for the codon positions for the protein-coding regions of Tmo-4c4 and the exons of aldolase and S7, and the protein-coding regions and tRNA segments of ND4 (partitions given in the electronic supplementary material, file S2). One thousand replicate ML inferences were performed and clade confidence was assessed with 1000 bootstrap pseudo-replicates employing the thorough bootstrapping algorithm (option ‘-b’). The analyses were rooted with *Syngnathoides biaculeatus*, with the three *Solegnathus* species unconstrained. Sequence matrices and resulting trees were deposited on TreeBASE (S15971).

### 3.6 Morphology and skeletal structure

Morphometric measurements were taken of the holotype and three paratypes, and compared with scaled photographs from field observations of leafy (*n*=25) and common seadragons (*n*=20; for details, see electronic supplementary material, file S1). The descriptive morphology follows [4], and proportional measurements were taken as in reference [28].

Micro-computed tomography (μCT) of the holotype was conducted at the Cartilage Tissue Engineering laboratory at the University of California San Diego. The specimen was imaged in a Skyscan 1076 (Kontich, Belgium), wrapped in ethanol-soaked gauze, and positioned in a sealed LDPE container. Imaging was conducted at 36 μm isotropic voxel size, applying an electrical potential of 100 kVp and a current of 100 μA, with a 1.0 mm aluminium filter. A beam-hardening correction algorithm was applied during image reconstruction. The reconstructed file size was 1000×1000×5482 pixels (*xyz*).

DICOM stacks were processed and segmented in Amira v. 5.4 (Visage Imaging, Inc.), and visualized in Maya 2014 service pack 4 (Autodesk, Inc.). X-ray radiographs of all seadragon species were obtained at Scripps Institution of Oceanography.

## 4. Results

### 4.1 Genetic distance

The new species showed clear genetic divergences in mitochondrial and nuclear sequences of a comparable magnitude to those between the established species of seadragons, both in uncorrected and model-corrected distances. As an example, the mitochondrial markers of the new species differed by 7.4% to *Phyllopteryx taeniolatus* and by 13.1% to *Phycodurus eques*, which is similar to the 11.6% distance between the two known species (uncorrected pairwise distances). Model-corrected distances (GTR + G) of the mitochondrial data showed 9.9% divergence between the new species and *Phyllopteryx taeniolatus*, and 22.3% to *Phycodurus eques*, similar to the two known species that diverged by 18.9%. Comparisons for the three nuclear loci showed a similar pattern (electronic supplementary material, tables S2–S4). The mitochondrial haplotype of the male was 6 bp different from its brood, which represented the mother's mitochondrial haplotype. The father and his offspring were genetically identical for the three nuclear markers.

### 4.2 Phylogenetic placement

All analyses retrieved the seadragons as a well-supported clade with respect to *Syngnathoides biaculeatus* and Solegnathinae (figure 1; for individual gene trees see electronic supplementary material, figure S3). Among seadragons, the new species was supported as the sister-species to the common seadragon, and the leafy seadragon as the sister to this clade (figure 1; electronic supplementary material, figure S3*a*,*c*,*d*). The nuclear S7 was incongruent with the other markers, supporting the leafy seadragon as the sister to the new seadragon (electronic supplementary material, figure S3*b*). MP and ML analyses gave similar topologies within the seadragons.

### 4.3 Morphology

**Figure 2.**
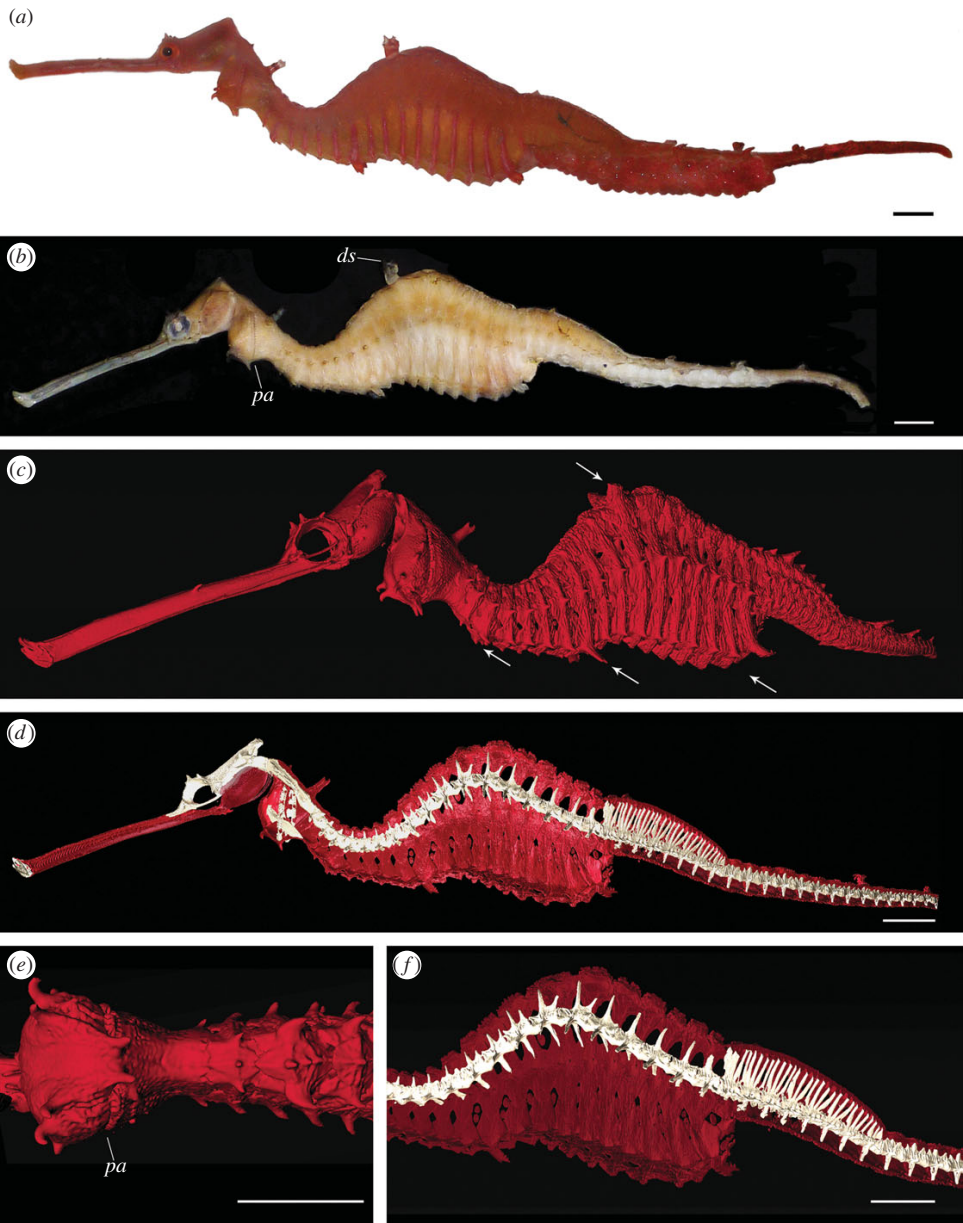
Holotype of the new seadragon *Phyllopteryx dewysea* n. sp. (*a*) On-deck shortly after being trawled; (*b*) preserved, with tip of tail and eggs removed for DNA extraction; *pa* pectoral area; *ds* facing dorsal spine; (*c*–*f*) three-dimensional scan generated by μCT; (*c*) outer bony plates, arrows pointing to different enlarged spines; (*d*) left half of the plates removed to reveal parts of the skull, pectoral girdle and spine (in white); (*e*) ventral view of the enlarged pectoral area; (*f*) detail of the trunk region. Scale bars, 1 cm.

**Figure 3.**
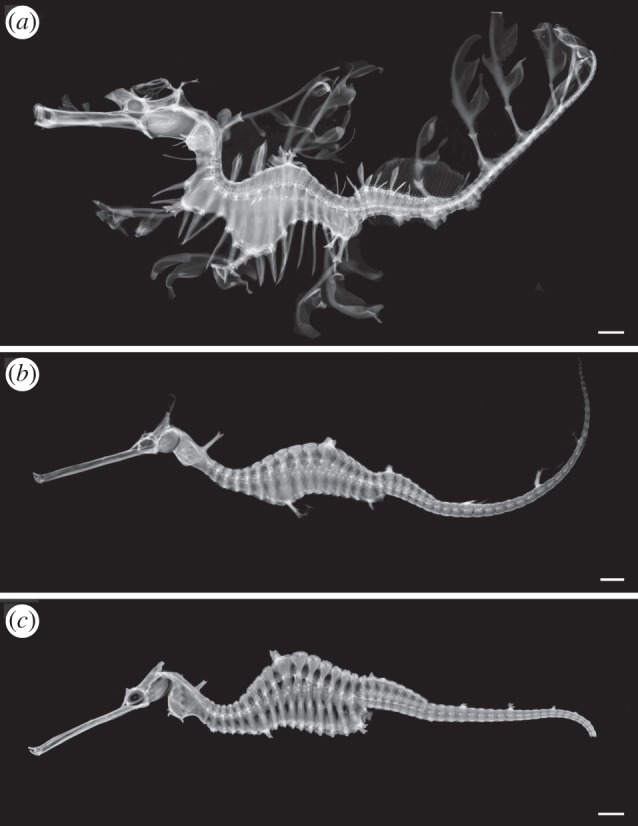
Comparison of the skeleton of the three species of seadragons. X-ray radiographs of (*a*) the leafy seadragon *Phycodurus eques*, SIO 04-28; (*b*) the common seadragon *Phyllopteryx taeniolatus*, SIO 84-300; (*c*) *Phyllopteryx dewysea* n. sp., holotype WAM P33223.002. Scale bars, 1 cm.

The new species has morphological features that clearly distinguish it from the other two seadragons (table 2; see also electronic supplementary material, file S1). In life, the holotype is easily distinguished by its red colour (figure 2*a*), and pink vertical bars that extend halfway up the body to the lateral trunk ridges (figure 2*b*). The more uniform coloration differentiates it from the common seadragon, which has numerous colourful spots and blotches (figure 1). These spots are always distinct, even in preserved specimens (see electronic supplementary material, figure S1) allowing differentiation of the two species even when the pigmentation is faded. The patterning also differs from the tan-yellow leafy seadragon, which has vertical bars that extend all the way up the body and are white with purple rims (figure 1). The X-ray radiographs and μCT showed that the new species has 18 trunk vertebrae with the corresponding outer bony plates (see electronic supplementary material, videos S1 and S2). Leafy seadragons have variable ring counts between 17 and 18. All common seadragons examined had only 17 trunk rings. The body shape of the new seadragon is reminiscent of the common seadragon, and different from the wave-like body of the leafy seadragon (figure 3). Morphometric measurements, however, did not reveal any perfectly diagnostic characters (table 3). The new species also has an enlarged pectoral area compared to the other species (*pa* in figure 2*b*,*e*). Even in large-bodied common seadragons (electronic supplementary material, figure S1*d*), the area is not as inflated as in the new species. In contrast to the backwards-facing pair of dorsal spines in common and leafy seadragons, the new seadragon has a forward-pointing pair (*ds* in figure 2*b*).

**Table 2.** Comparison of diagnostic traits among the three seadragon species.

	*Phycodurus eques* (*n*=25)	*Phyllopteryx taeniolatus* (*n*=20)	*Phyllopteryx dewysea* n. sp. (*n*=4)
trunk rings	17–18	17	18
head spine	present	present	absent
third ventral spine on trunk ring	16	16 or absent	17
dorsal spine on trunk ring 11 orients	backward (figure 3*a*)	backward (figure 3*b*)	forward (figure 2*b*, figure 3*c*)
coloration	yellow/pink and white bars	yellow spots and blue ridges	red/pink bars
dermal appendages	complex, multilobate	simple, spatulate	unknown
lateral trunk and lateral tail ridge	continuous	discontinuous	discontinuous

**Table 3.** Morphometric measurements for the three seadragon species. Values for *Phyllopteryx dewysea* are given separately for the holotype and as ranges for the three paratypes. Proportions are given as % of the total length (TL), head, or snout length. The tail length of the holotype was measured from the photograph (figure 1*a*) as the tail is incomplete in the preserved specimen.

	*Phycodurus eques* (*n*=25)	*Phyllopteryx taeniolatus* (*n*=20)	*Phyllopteryx dewysea* n. sp. (holotype | three paratypes)
total length (TL, mm)	218–401	238–427	240 | 221–259
trunk length (mm)	67–114	70–135	67 | 61–79
tail length (mm)	114–221	98–234	111 | 92–123
proportions (%)			
trunk length : TL	25–42	24–34	28 | 28–31
tail length : TL	45–69	42–58	46 | 41–48
head length : TL	18–33	17–29	26 | 22–31
snout length : head length	53–77	61–72	68 | 65–74
snout depth : snout length	11–20	7–13	7 | 7–10
orbital diameter : head length	10–13	8–13	12 | 10–13
postorbital length : head length	23–36	11–28	20 | 18–24
neck depth : TL	4–11	3–7	5 | 3–4
trunk depth 8th segment : TL	8–18	4–14	8 | 7–9
trunk depth 11th segment : TL	8–17	6–13	11 | 9–12
trunk depth preanal segment : TL	6–12	4–9	9 | 6–8

### 4.4 Systematics

*Phyllopteryx dewysea*n. sp. (figure 1, figure 2*a*–*f*, figure 3*c*, tables 2 and 3, electronic supplementary material figure S2*a*,*b* and videos S1–S2).

#### 4.4.1 Holotype

The holotype repository is the Western Australian Museum, Perth: WAM P33223.002, as *Phyllopteryx taeniolatus*, 240 mm TL, male with brood attached; eggs removed for DNA extraction, tail clipped. The species has been registered with Zoobank under the following LSID: BEF4C635-D5DD-4F70-99B6-88157AA88C7C.

#### 4.4.2 Type locality

Australia, Western Australia, Recherche Archipelago, east of Middle Island, trawled at 51 m, Marine Futures Survey 2007, station MF-MI-007, 10 October 2007, 34°01.589^′^0 S, 123°21.55^′^0 E to 34°01.30^′^ S, 123°21.42^′^ E.

#### 4.4.3 Paratypes

Three paratypes are designated based on morphological similarity to the holotype (table 3 and electronic supplementary material, file S1). CSIRO Marine and Atmospheric Research, ANFC C2269 and C2270, as *Phyllopteryx lucasi* (=*taeniolatus*), two specimens, 236 and 259 mm TL, sex undetermined; trawled west of Garden Island, Western Australia, 32° S, 115° E (coordinates approximated), 72 m, May 1956.

WAM P660, as *Phyllopteryx foliatus,*one specimen, 221 mm TL, sex undetermined; beachwashed onto Cottesloe Beach, Western Australia, 31°59^′^ S, 115°45^′^ E, August 1919.

#### 4.4.4 Etymology

Named for Mary ‘Dewy’ Lowe, for her love of the sea and her support of seadragon conservation and research, without which this new species would not have been discovered.

#### 4.4.5 Diagnosis

Eighteen trunk segments. Head crest without appendage. Pectoral area enlarged (*pa* in figure 2*b*,*e*). Dorsally enlarged spine on trunk ring 11 pointed forward (*ds* in figure 2*b*). Paired enlarged ventral spines on rings 8 and 17 (figure 3*c*). Body deepest on ring 12, behind dorsal spine. Spines on lateral trunk ridge not continuous with lateral tail ridges (figure 2*c*).

#### 4.4.6 Brief description of holotype

Preserved colour light brown. Live colour ruby red, with pink vertical bars on each trunk segment and light markings on the snout (figure 2*a*). Enlarged spines dorsally on neck and trunk ring 11; ventrally on rings 3, 8, 17 (arrows in figure 2*c*; see also videos S1–S2 included in the electronic supplementary material). Presence and shape of dermal appendages on enlarged spines unknown. Lateral trunk ridges not confluent with lateral but with inferior tail ridges. Trunk dorsally forming pronounced arch, highest point ring 12, behind enlarged dorsal spines (figure 3*c*). Fin rays: pectoral 22; dorsal unknown; anal 4.

#### 4.4.7 Distribution and bathymetry

The type locality (arrow in map of figure 1) at 50+ m shows a complex habitat of a mixed reef and sandy habitat [29,30]. Two paratypes were collected from the continental shelf near Perth (72 m, circle in figure 1); the habitat at this site is unknown. The fourth paratype, collected at Cottesloe, Perth, does not have depth data, as it was washed up on the beach.

#### 4.4.8 Remarks

The placement of this species within *Phyllopteryx* was informed by the phylogeny of seadragons (figure 1). The new species lies as the sister group to *Phyl. taeniolatus* and this does not allow for its inclusion within the other seadragon genus, *Phycodurus*. We made the decision to place it within *Phyllopteryx* rather than erect a new genus, as this provides information about the closer relationship of *Phyl. dewysea* with *Phyl. taeniolatus* than with *Phyc. eques*.

An expanded description of the type series of *Phyl. dewysea*, of *Phyl. taeniolatus* and *Phyc. eques*, including detailed comparisons, can be found in the electronic supplementary material, file S1.

## 5. Discussion

The discovery of a new species of seadragon represents an important finding. It is genetically distinct from the two known seadragons, with supporting evidence from morphology and habitat. Although the two previously known seadragon species were described in the nineteenth century, *Phyl. dewysea* remained unrecognized for almost a century, after being first collected in 1919. This seadragon may occasionally be washed ashore, and be subjected to trawling, but may not have been recognized (as was the case with the samples located for this study) owing to a superficial similarity to common seadragons. Nevertheless, it seems surprising that this species escaped recognition until now given the public interest in seadragons. A possible explanation is that it may occur in depths beyond recreational SCUBA range: three of our specimens of *Phyl. dewysea* came from relatively deep water (51 and 72 m) a few kilometres offshore. The bathymetric range of common and leafy seadragons is insufficiently known, but they seem to be mostly restricted to inshore areas [4,31]. Clearly, two sampling localities in offshore waters do not make *Phyl. dewysea* an exclusive deeper water species, but the coloration of the holotype may be explained by a deeper habitat: although the red colour is conspicuous when out of the water (figure 2*a*), red light is rapidly absorbed with depth and so being red may effectively render the seadragon cryptic [32].

Basic biological information on habitat preferences and distribution are needed to assess the status of *Phyl. dewysea* in the wild, and underpin any future management plans. The few records of the new seadragon from southwestern Australia that we currently have cannot give a satisfactory picture of the distributional range. Furthermore, the records off the coast of Perth are almost 60 years old and it is unclear if *Phyl. dewysea* still occurs in this region. The urban development surrounding the Perth region has been significant in the past century [33], including changes in water quality and vegetation [34,35] that may have also affected the offshore habitat of *Phyl. dewysea*.

Only the retention of the unrecognized *Phyl. dewysea*specimens in museum collections allowed for its discovery as a new species. This highlights the significance of natural history collections as a treasury of new species [36], even decades after their collection [37]. Another invaluable component of biodiversity discovery consists of exploratory expeditions such as the Marine Futures project that trawled the holotype and mapped its habitat [29]: the on-board documentation provided the only available photograph of a fresh *Phyl. dewysea*, a currently unique representation of its natural coloration (figure 2*a*).

Southwestern Australia hosts a large number of marine fish [38], and is considered a hotspot for future discovery [3]. *Phyl. dewysea* is an illustration of the surprises that are waiting in the ocean, but many open questions remain regarding its range, habitat and biology. The other two seadragons are considered Near Threatened by the IUCN [6,39], and their conservation is of public interest [5,40]. The new seadragon is no less charismatic and deserves to become a flagship species for the discovery of biodiversity.

## Supplementary Material

File S1. Systematics and taxonomy of the three seadragon species. Gene trees. Includes figure S1. The common seadragon Phyllopteryx taeniolatus; figure S2. Paratypes of Phyllopteryx dewysea; figure S3. Individual gene trees.

## Supplementary Material

File S2. Molecular data partitions.

## Supplementary Material

Table S1-S4. Genetic distances between the seadragon species for mitochondrial data (table S1) and nuclear data (table S2-S4).

## Data accessibility

DNA sequences: GenBank accessions KM201544–KM201604. Phylogenetic data: TreeBASE accession number S15971. http://purl.org/phylo/treebase/phylows/study/TB2:S15971.
