# Supplementary material for: A spectacular new species of seadragon (Syngnathidae)
Source: R Soc Open Sci. 2015 Feb 18;2(2):140458. doi: 10.1098/rsos.140458 (PMC4448810; doi:10.1098/rsos.140458)
Supplement: File S1. Systematics and taxonomy of the three seadragon species. Gene trees. Includes figure S1. The common seadragon Phyllopteryx taeniolatus; figure S2. Paratypes of Phyllopteryx dewysea; figure S3. Individual gene trees. [file rsos140458supp1.docx]

**ELECTRONIC SUPPLEMENTARY MATERIAL**

# A spectacular new species of seadragon (Syngnathidae)

Josefin Stiller, Nerida G. Wilson, and Greg W. Rouse

**File S1**. Systematics and taxonomy of the three seadragon species. Gene trees.

**Figure S1**. The common seadragon *Phyllopteryx taeniolatus*.

**Figure S2**. Paratypes of *Phyllopteryx dewysea*.

**Figure S3**. Individual gene trees.

*Systematics*

*Phycodurus eques,* leafy seadragon

*Phyllopteryx*

*Phyllopteryx taeniolatus,* common seadragon

*Phyllopteryx dewysea* n. sp.

Expanded description of holotype

Paratypes and morphological variation

Comparisons

The abbreviations refer to the following ichthyological collections:

AMS Australian Museum, Sydney

ANFC CSIRO Marine and Atmospheric Research, Australian National Fish Collection, Hobart

SIO Marine Vertebrate Collection, Scripps Institution of Oceanography, San Diego

SAM South Australian Museum, Adelaide

WAM Western Australian Museum, Perth

CAS California Academy of Sciences, San Francisco

MHNH Muséum National d'Histoire Naturelle de Paris

BMNH British Museum of Natural History, London

# *Phycodurus* Gill, 1896

*Phycodurus* Gill, 1896:159 (type species by original designation: *Phyllopteryx eques* Günther, 1865); Dawson, 1985:157; Paxton et al., 1989:427; Kuiter, 2000:80; Paxton et al., 2006:838.

## *Phycodurus eques* (Günther, 1865)

(figure 1, figure 3*a*, table 2-3)

Whitley & Allan, 1958:48, pl. 1, fig. 11; Dawson, 1985:158, fig. 236-237; Paxton et al., 1989:427-428; Kuiter, 2000:80-87, figs. A-Z2; Paxton et al., 2006:838-839.

*Synonymy.*

*Phyllopteryx eques* Günther, 1865:327, pl. 15 (Port Lincoln, South Australia; holotype: BMNH 1865.5.13.2); McCulloch, 1929:95; Gomon et al. 1994:462-463, figs. 414a-b.

*Phycodurus glauerti* Whitley, 1939:268 (Rottnest Island, Western Australia; holotype: AMS IA.7596).

*Common names*. Leafy seadragon, Glauert’s seadragon.

*Material examined.*

25 adult individuals from Western Australia (10 from Bremer Bay; of those 5 pregnant males, 5 of undetermined sex) and South Australia (3 from Rapid Bay, 4 from Encounter Bay, 8 from Wool Bay; all of undetermined sex); measurements taken on scaled photographs.

SIO 04-28, *Phycodurus eques*, 1 specimen, 275 mm TL. Reared by commercial aquarium supplier, died at Birch Aquarium, 9 March 2004.

AMS IA.7596, *Phycodurus glauerti*, holotype. Photograph. Collected near Rottnest Island, Western Australia by the Western Australian Museum, 1938.

*Diagnosis*.

Color in life tan-yellow with pink and white bars extending between the inferior to the superior trunk ridges (figure 1). 17 – 18 trunk segments. Enlarged spines carry multilobate dermal appendages. Head crest with enlarged spine. Pectoral area not enlarged. Dorsally enlarged spine on trunk segment 11 pointed backwards (figure 3*a*). Paired enlarged ventral spines on trunk rings 8 and 16, seldom on 17. Thorax curved wavelike. Spines on the lateral trunk ridge continuous with the lateral and superior tail ridges.

*Description.*

Large dermal appendages on head, neck, trunk, and tail, of tan-yellow color with dark spots (figure 1). Head with white and dark bars crossing the eye and extending to preopercular region and on snout; pronounced wedge-like frontal ridge (figure 3*a*). One large orbital spine above eye with blunt end. Pectoral area only slightly inflated, bearing a small number of elongate threadlike spines. Unpaired head crest sharply triangular, distally with delicate, multilobate dermal appendage. Unpaired neck spine present, with broad base, distally with complex, multilobate dermal appendage. Inferior trunk ridges with threadlike protuberances on each segment, except conspicuously enlarged spines on trunk rings 3, 8, each with a well-developed large multilobate dermal appendage; preanal multilobate dermal appendages on ring 16 (occasionally on 17), Superior trunk ridges with blade-shaped spines on several segments, largest on trunk ring 11, dorsally with a pair of broad-based large spines (occasionally on ring 12) each bearing a complex, multi-lobed appendage; spines pointing backwards. Median ventral trunk ridge with unpaired blade-shaped spines. Lateral trunk ridge with threadlike spines on each segment; towards tail confluent with lateral tail ridges and with superior tail ridges. Trunk undulating wave-like (figure 3*a*); dorsally forming a pronounced arch, highest point around trunk ring 10-12, near the base of the enlarged dorsal spines; ventrally with three pronounced bulges, near the bases of the enlarged ventral spines. Number of fin rays pectoral 20; dorsal 36-37; anal 4. Tail with 43 segments.

*Remarks*. Distinguishing between the sexes is difficult in this species unless the males carry eggs. The morphometric measurements for this species (table 3) are therefore based mostly of species of undetermined sex.

The literature reports 18 trunk rings for this species [1–3]. We also observed mostly 18 trunk segments but occasional cases of 17 segments. While the large trunk rings can be reliably counted on preserved specimens and photographs, the small rings of the tail can be difficult to count towards the tip (Figure 3*a*). We therefore only counted the 43 tail segments in the X-rayed individual that falls within the range reported in the literature (36 - 44 tail rings, [1–3]).

# *Phyllopteryx* Swainson, 1839

*Phyllopteryx* Swainson, 1839:332 (type species by original designation: *Syngnathus foliatus* Shaw, 1804 (= *syngnatus* [sic] *taeniolatus* Lacépède, 1804); Castelnau, 1872:199; Dawson, 1985:158-160; Paxton et al., 1989:428; Kuiter, 2000:75; Paxton et al., 2006:839.

## *Phyllopteryx taeniolatus* (Lacépède, 1804)

(figure 1, figure 3*b*, figure S1*a-e*, table 2-3)

Whitley & Allan, 1958:45-48, text-fig. 11, 1 (as *Phyllopteryx lucasi* from Kangaroo Island, South Australia); pl. 1, fig. 8; Dawson, 1985:160, fig. 238; Kuiter, 1993:95, 4 figs; Paxton et al., 1989:428; Gomon et al. 1994:463-464, figs. 415a-b; Kuiter, 2000:75-79, figs. A-W; Paxton et al., 2006:839.

*Synonymy.*

*syngnatus* [sic] *taeniolatus* Lacépède, 1804:211, pl. 58, fig. 3.; 203-204 and footnote (2) on 204-205 (Bass Strait; type unknown).

*Syngnathus foliatus* Shaw, 1804:456, pl. 180 (‘New Holland’; type unknown); Perry, 1811:2 unnumbered pages, 1 unnumbered plate (as *Syngnathus* or *Hippocampus foliatus*).

*Phyllopteryx foliatus* McCulloch, 1929:95; Castelnau, 1872a:198-199; McCoy, 1882:19-20, pl. 65, fig. 1a-b.

*Syngnathus taeniopterus* Günther, 1865:327 (locality unknown; type unknown).

*Phillopteryx* [sic] *elongatus* Castelnau, 1872b:243 (Gulf St. Vincent, South Australia; types: MNHN 728, 834-5, 9227).

*Phillopteryx* [sic] *elongatus* Castelnau, 1873:144 (*non Phillopteryx elongatus* Castelnau, 1872b; Fremantle, Western Australia).

*Phyllopteryx altus* McCoy, 1882:20 (description based on drawings of plate 14 of Günther, 1865 and plate 7 of Meredith, 1880; presumably from Tasmania).

*Phyllopteryx lucasi* Whitley, 1931:102-103, pl. 15(1) (Holotype: AMS IA.4119; Middleton Beach, Albany, Western Australia); Whitley & Allan, 1958:48 (not the specimen depicted in text-fig. 11, 1)

*Common names*. Common, Lucas’ or weedy seadragon.

*Material examined.*

20 males from Western Australia (9 from Esperance, 1 from Albany) and Tasmania (3 from Bicheno, 4 from Hobart, 3 from Blackmans Bay); measurements taken on scaled photographs.

AMS I.17721-001, *Phyllopteryx taeniolatus*, 1 specimen. Collected by prawn trawl in Newcastle, Sydney Basin, New South Wales, 32°56’S, 151°46’E by J.J. Bennet, January 1974.

AMS I.12376, *Phyllopteryx taeniolatus*, 9 specimens. Collected in Investigator Strait and south of Kangaroo Island, South Australia, 35°25’S, 137°22’E on FIV *Endeavour*, January 1912.

AMS I.21307-002, *Phyllopteryx taeniolatus*, 1 specimen, 262 mm TL. Collected by bottom trawl east of Chappel Island, Furneaux Group, Tasmania, 40°17’S, 148°01’E on FRV *Challenger*, 14 October 1979.

CAS SU31883, *Phyllopteryx foliatus*, 1 specimen. Collected near Perth, Western Australia by the Western Australian Museum, 31 May 1996.

SAM A2018, *Phyllopteryx taeniolatus*, 1 specimen. Collected by trawl near Evans Island, Ceduna, South Australia, 32°07’60S, 133°42’E on prawn trawl *Beaver*, 20 April 1982.

SIO 73-361, *Phyllopteryx foliatus*, 1 specimen, ca. 330 mm TL, female. Collected in Tasmania via T.H. Bullock, August 1973.

SIO 84-300, *Phyllopteryx taeniolatus*, 1 specimen, 258 mm TL, subadult male. Collected in Eagle Bay, Geographe Bay, Western Australia, 33°33’ S, 115°04’E in 1-5 meters by B.C. Russell, 1 April 1978.

WAM P.14847, *Phyllopteryx taeniolatus*, 1 specimen. Collected in Shoalwater Bay, Western Australia, 32°17’S, 115°41’E by L. Harris, 15 January 1966.

WAM P.20854, *Phyllopteryx taeniolatus lucasi*, 1 specimen, 219 mm TL. Collected by trawl 5 ½ miles west of Garden Island, 32°13.3’S, 115°33.32’E to 32°13.01’S, 115°31.05’E in 27-33 meters on FRV *Flinders*; collected by L. Marsh and M. Shepherd, 8 March 1972.

WAM P.29073, *Phyllopteryx taeniolatus*, 1 specimen, 270 mm TL (tail incomplete). Collected near Garden Island, Western Australia, 32°15’S, 115°40’E by R.B. Humphries, 11 June 1972.

*Diagnosis*.

Color in life purple-brown with lateral yellow spots and first trunk rings with purple bars extending between the inferior and lateral trunk ridges (figure 1). Enlarged spines carry spatulate appendages. 17 trunk segments. Head crest with enlarged spine. Pectoral area not enlarged. Dorsally enlarged spine on trunk segment 11 pointed backwards. Paired enlarged ventral spines on trunk ring 8, each with spatulate dermal appendages; preanal dermal spatulate appendage on ring 16 often absent or only small. Body deepest on trunk segments 10-11, before the dorsal spine (figure 3*b*). Spines on the lateral trunk ridge not continuous with the lateral tail ridges.

*Description.*

Elongate dermal appendages on head, neck, trunk, and tail of dark color, often with black border, some with yellow markings (figure 1). Preserved color faint brown, with spots and blotches visible (figure S1*a*, *c*). Head with yellow spots on preopercular region and white spots on snout (figure S1*d-e*); frontal ridge inconspicuous. One large orbital spines above eye with pointed end, slightly pointing backward. Pectoral area only slightly inflated, bearing a number of small spines (figure S1*d-e*). Unpaired head crest sharply triangular, elongate and often with yellow spots, distally with elongate dermal appendage. Unpaired neck spine present, narrow and elongate, often with white spots, distally with spatulate dermal appendage. Inferior trunk ridges with small spines on each segment, except conspicuously enlarged spines on trunk rings 3, 8, and 16: ring 3 with small backwards facing spines surrounded by dark purple coloration in living animals; ring 8 with enlarged thin spines each carrying a spatulate dermal appendage; ring 16 with either small backwards facing spines without dermal appendages (arrow in figure S1*c*) or enlarged spines with small spatulate dermal appendages (arrow in figure S1*a*; figure S1*b*). Superior trunk ridges with minute protuberances on each segment, except for conspicuously enlarged spines on trunk ring 11, often with spots, each bearing a spatulate dermal appendage; spines pointing backwards. Median ventral trunk ridge with minute spines. Lateral trunk ridges with small spines on each segment; towards tail not confluent with lateral tail ridges but with inferior tail ridges. Trunk smoothly curved (figure 3*b*); dorsally forming a pronounced arch, highest point around trunk ring 10-11, before the base of the enlarged dorsal spines; ventrally with two smooth bulges, near the bases of the enlarged ventral spines on segment 8 and 16. Number of fin rays pectoral 20-21; dorsal 30-32; anal 4. Tail with 31 to 37 segments.

*Remarks*. This species was first described by the French naturalist Counte Étienne Lacépède in May 1804 from a specimen that was collected by the explorer Baudin in Bass Strait. No holotype was designated and it remains unknown if the type was collected on the coast of Victoria or Tasmania or on one of the islands in Bass Strait.

The literature reports 17 to 18 trunk segments for this species [1–3]. However, we consistently observed 17 segments in all examined material. It remains to be established if this trait is polymorphic in the common seadragon like we have observed here for the leafy seadragon (see above). It is also possible that the literature value of 18 trunk segments was based on specimens of *Phyl. dewysea* that were not recognised as distinct.

Common seadragons are sexually dimorphic with females having a deeper trunk compared with the males (compare figure S1*a* to S1*c*). The morphometric measurements presented in table 2 are only based on male common seadragons in order to allow for better comparability to *Phyl. dewysea*.

***Phyllopteryx dewysea* n. sp.**

(figure 1, figure 2*a-f*, figure 3*c*, figure S2*a-d*, table 2-3, videos M1-2)

*Expanded description of holotype.* In life ruby red, each trunk segment with a pink vertical bar extending between the inferior and lateral trunk ridges, snout with light markings (figure 2*a*). Preserved color light brown, with vertical bars pale pink (figure 2*b*); frontal ridge inconspicuous. Two small orbital spines above eye with pointed end, anterior one slightly pointing forward, posterior one pointing outward (see video M1). Pectoral area (*pa* in figure 2*b*) greatly inflated, with several conspicuous hook-like spines (figure 2*e*). Unpaired head crest triangular, bears no signs of a broken dermal appendage. No dermal appendages observed on enlarged spines. Unpaired neck spine present, narrow and elongate, appears distally broken. Inferior trunk ridges with small spines on each segment, except conspicuously enlarged spines on trunk rings 3, 8, and 17 ventrally with a pair of enlarged spines (arrows in figure 2*c*): ring 3 with backwards facing spines; ring 8 with enlarged thin spines, appear distally broken; ring 17 with enlarged thin spines, appear distally broken. Superior trunk ridges with small protuberances on each segment, except for conspicuously enlarged spines on trunk ring 11, appear distally broken; spines pointing forward (arrow in figure 2*b*). Median ventral trunk ridge with minute spines. Lateral trunk ridges with backwards-facing spines on each segment; towards tail not confluent with lateral tail ridges but with inferior tail ridges. Trunk smoothly curved; dorsally forming a pronounced arch, highest point around trunk ring 12, behind the base of the enlarged dorsal spines; ventrally with two indistinct bulges, near the bases of the enlarged ventral spines on segment 8 and 17. Fin rays: pectoral 22; dorsal unknown; anal 4. Tail incomplete, 22 segments left.

*Paratypes and morphological variation*

ANFC C2269 and C2270, *Phyllopteryx lucasi (=taeniolatus),* 2 specimens, 236 and 259 mm TL, sex undetermined. Trawled west of Garden Island, Western Australia, 32°S, 115°E (coordinates approximate) in 72 m, May 1956.

WAM P660, 1 specimen, 221 mm TL, sex undetermined. Collected at Cottesloe, Western Australia by Lt. Comm. Barlee, 31°59’S, 115°45’E, beach-washed, August 1919.

*Variation.* The paratypes closely match to the description of the holotype and show all the diagnostic features of the holotype. Only variations and additions are being listed here: Posterior orbital spine of equal size as anterior one, or smaller. Number of fin rays pectoral 21-23; dorsal 31; anal 4. Tail with 41 – 42 segments.

We currently do not have enough information to determine the sex of the new seadragon based on external features. The sex of the holotype was obvious since it carried eggs but none of the paratypes carried a brood. Morphometric values are similar between the male holotype and the paratypes (table 2) and no external features were observed that would allow us to infer the sex. We refrained from dissection of the types until more specimens are found.

*Comparisons*

*Phyllopteryx dewysea* n. sp. has a number of characters that are shared with either the leafy or the common seadragon, in addition to apomorphies that distinguish it from its relatives (table 1).

*Colouration and patterning*. In life, *Phyl. dewysea* is distinguished by its red colour with pink bars (figure 2*a*). The other two seadragons each display a characteristic patterning: The common seadragon has light spots and purple bars, and the leafy seadragon has purple-rimmed white bars on a yellow-green body (figure 1). These patterns fade in alcohol but are still clearly distinguishable (figure S1*a, c*). The trunk segments of *Phyl. dewysea* have pink bars that extend halfway up the body, from the lateral trunk ridges to the superior trunk ridges. They are similar to the ones in the leafy seadragon but in that species they extend all the way up to the superior trunk ridges.

*Body shape*. *Phyllopteryx dewysea* has a body shape that is similar to the common seadragon with a broad dorsal arch that is different from the leafy seadragon with its dramatically wavelike trunk (see figure 3*a-c*). The organization of this dorsal arch differs between the members of *Phyllopteryx*: In *Phyl. dewysea* the deepest part of the body is on segment 12 and therefore located behind the dorsal enlarged spine (figure 2*b*; figure 3*c*). In *Phyl. taeniolatus* on the other hand, the deepest segment is on segment 10 or 11, before or on the segment of the enlarged spine (figure 3*b*; figure S1*a*, *c*).

*Trunk segments*. The new species has 18 trunk rings, which distinguishes it from the common seadragon with its 17 trunk rings. The trunk ring count in leafy seadragons is mostly 18, but individuals with only 17 segments were observed. Given the phylogenetic relationships (figure 1), a trunk ring count of 18 seems could have been the ancestral condition in seadragons or could have evolved independently in *Phyc. eques* and *Phyl. dewysea*.

*Enlarged spines and dermal appendages.* The enlarged spines of the inferior and superior trunk ridges are characteristic of all three seadragons. In the common and the leafy seadragon, the enlarged spines carry dermal appendages. We suspect that the new seadragon also carries some kind of dermal appendages but cannot ascertain any shape, as the available specimens had only the enlarged spines (arrows in figure 2*b-c*) without the dermal appendages. All three species have enlarged spines ventrally on the third trunk ring, small and inconspicuous in the new species and in the common seadragon (figure 3*b-c*), but enlarged and with leaf-like dermal appendages in the leafy seadragon (figure 3*a*). Another enlarged spine is located dorsally on trunk segment 11 in all species. It points backwards in the leafy and common seadragons but forward in *Phyl. dewysea*. The orientation in the common seadragon is similar between males and females (compare figure S1*a, c*) and ontogenetic changes in this trait were not observed in juvenile to subadult seadragons (JS, personal observation on captive common and leafy seadragons at Birch Aquarium, UCSD). As part of ongoing projects on seadragons, we have photos of ca. 180 common seadragons and ca. 30 leafy seadragons (Wilson et al. unpub.) and there are no signs of ontogenetic or sex-specific differences.

The new seadragon has an enlarged spine ventrally, located in front of the anal segment, which corresponds to the 17^th^ trunk ring. In leafy seadragons, the corresponding enlarged spine is located on segment 16 or 17, and on segment 16 in common seadragons. In common seadragons, the spine on this segment can have different forms, from being entirely reduced, to a small spine lacking a dermal appendage in most specimens observed (arrow in figure S1*c*), to an enlarged spine carrying a small dermal appendage (arrow in figure S1*a*; detail figure S1*c*). The area surrounding the spine is often of a conspicuous dark purple coloration in common seadragons (figure 1), which is absent in the new species and in leafy seadragons.


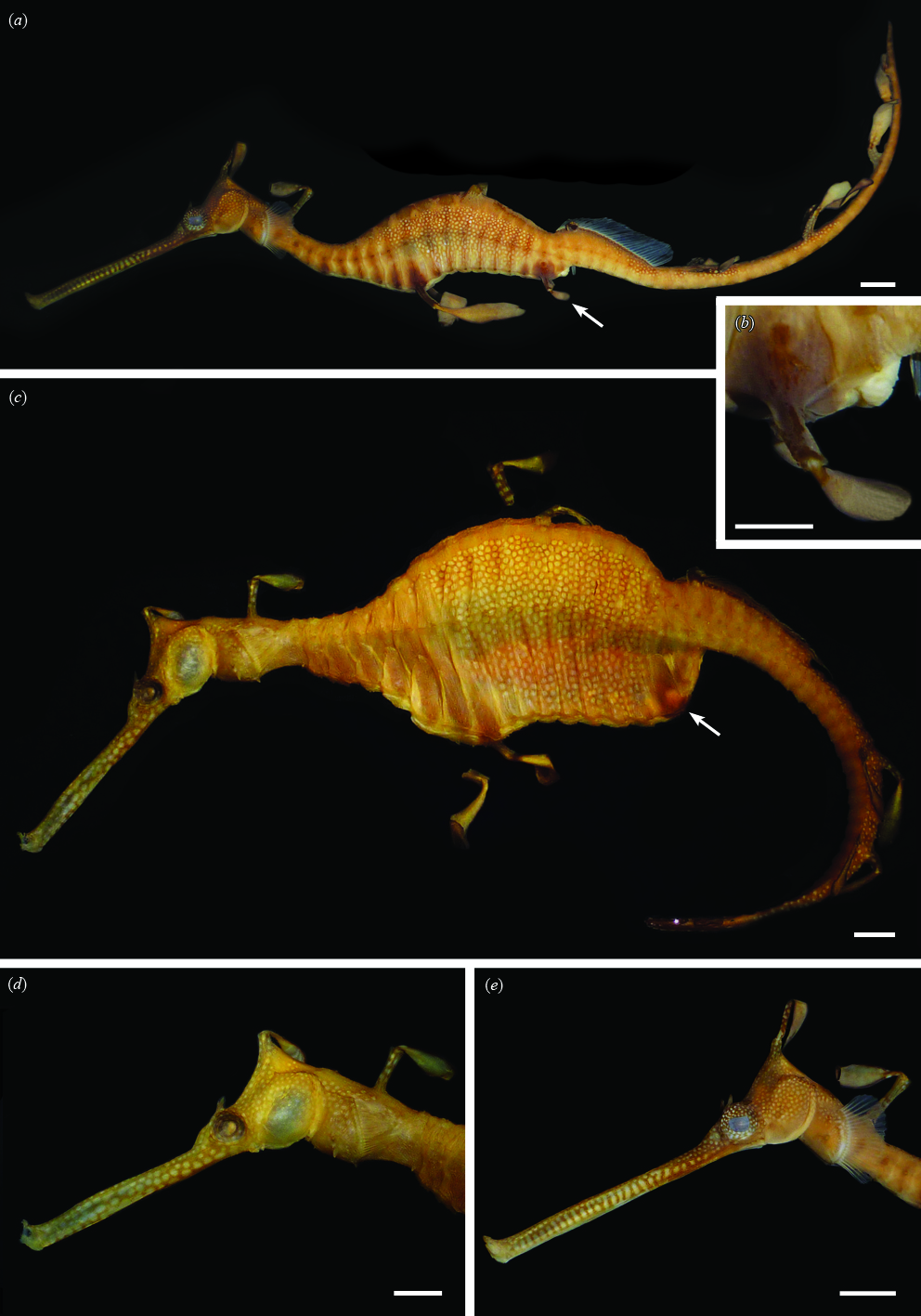


**Figure S1.** The common seadragon *Phyllopteryx taeniolatus*, (*a-b, e*) subadult male, SIO 84-300, (*a*) habitus, arrow points to well-defined spine and dermal appendage on preanal segment 16, (*b*) detail of the preanal appendage, (*e*) head region. (*c, d*) adult female, SIO 73-361, (*c*) habitus, arrow points to the preanal 16^th^ segment that lacks an enlarged spine and dermal appendage, note that two dorsal dermal appendage are not attached, (*d*) head region. Scale bars 1 cm.


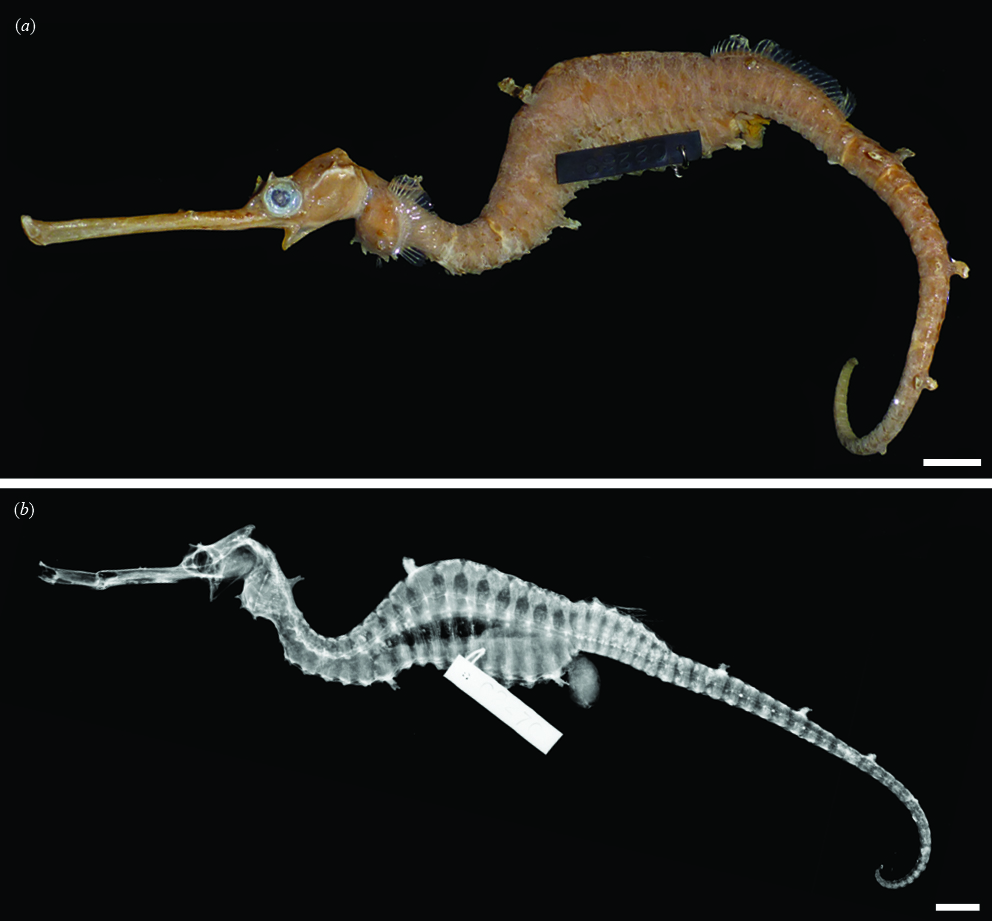


**Figure S2**. Two of the paratypes of *Phyllopteryx dewysea*, n. sp., (*a*) preserved specimen, ANFC C2269, (*b*) radiograph showing of the skeleton, ANFC C2270. Note the enlarged pectoral area and the forward-facing dorsal spine. Scale bars 1 cm.


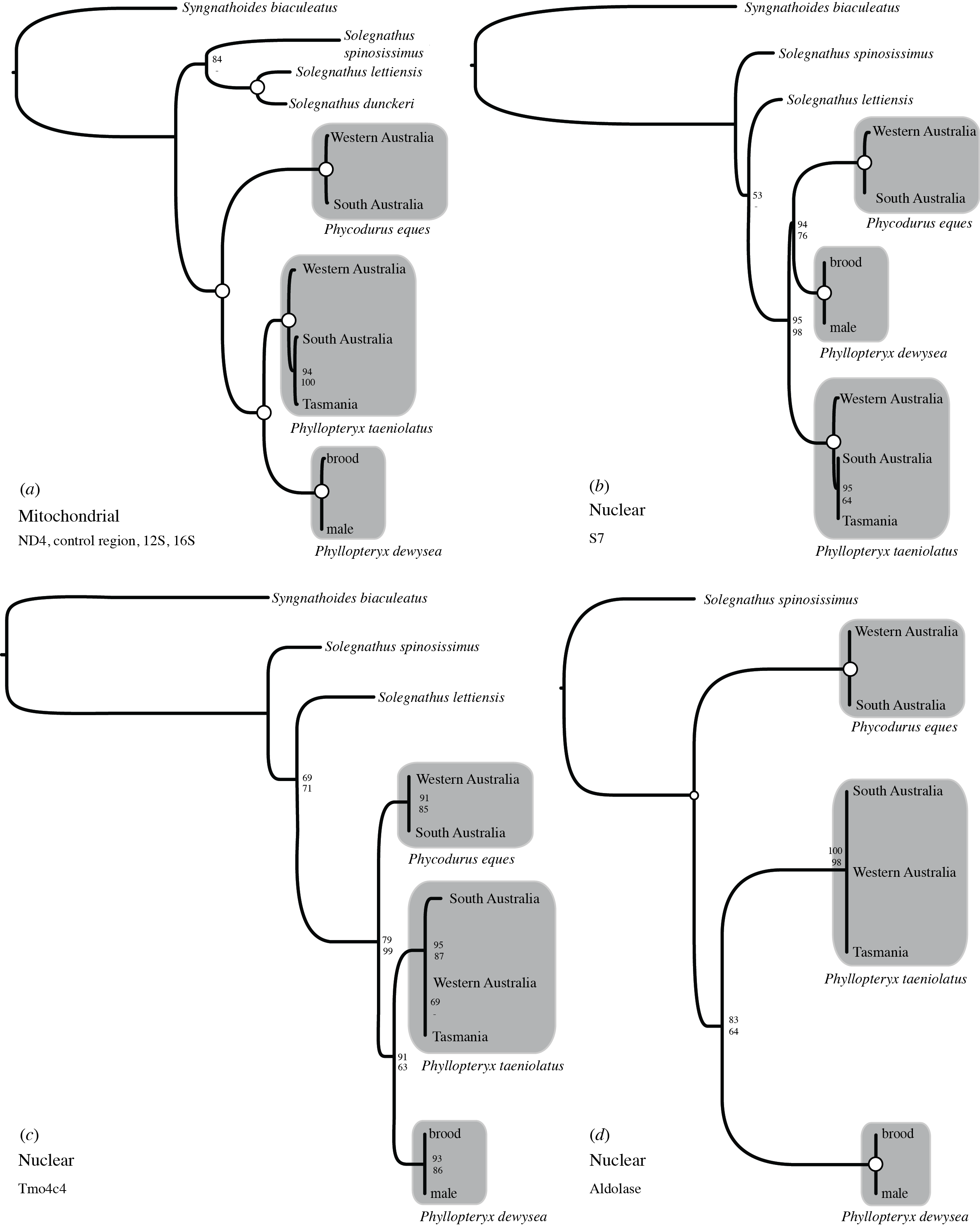
**Figure S3**. Maximum likelihood reconstruction of the phylogenetic relationships of the three seadragon species based on the individual datasets, (*a*) mitochondrial markers (2790 bp), (*b*) S7 ribosomal protein gene (685 bp), (*c*) Tmo-4c4 gene (559 bp), (*d*) Aldolase-like gene (382 bp). Numbers next to nodes indicate bootstrap and jackknife support >50 %; white circles on nodes reprsent 100 % bootstrap and 100 % jackknife support.

**Supplementary References**

1. Kuiter, R. H. 1993 *Coastal Fishes of South-Eastern Australia*. Honolulu: University of Hawaii Press.

2. Dawson, C. E. 1994 Syngnathidae. In *The Fishes of Australia’s South Coast* (eds M. F. Gomon J. C. M. Glover & R. H. Kuiter), pp. 440–475. Adelaide: State Print.

3. Dawson, C. E. 1985 *Indo-Pacific Pipefishes (Red Sea to the Americas)*. Gulf Coast Research Laboratory.
