# Supplementary material for: A spectacular new species of seadragon (Syngnathidae)
Source: R Soc Open Sci. 2015 Feb 18;2(2):140458. doi: 10.1098/rsos.140458 (PMC4448810; doi:10.1098/rsos.140458)
Supplement: File S2. Molecular data partitions. [file rsos140458supp2.docx]

**ELECTRONIC SUPPLEMENTARY MATERIAL**

# A spectacular new species of seadragon (Syngnathidae)

Josefin Stiller, Nerida G. Wilson, and Greg W. Rouse

**File S2**. Molecular data partitions.

Partitions for Aldolase-like gene:

DNA, Ald-1= 2-113\3, 367-382\3

DNA, Ald-2 = 3-114\3, 368-380\3

DNA, Ald-3 = 1-115\3, 369-381\3

DNA, Ald-intron = 116-366

Partitions for S7 ribosomal protein gene:

DNA, S7-intron = 1-649

DNA, S7-2 = 650-683\3

DNA, S7-1 = 651-684\3

DNA, S7-3 = 652-685\3

Partitions for Tmo-4c4 gene:

DNA, Tmo-1= 1-559\3

DNA, Tmo-2 = 2-557\3

DNA, Tmo-3 = 3-558\3

Partitions for the combined nuclear data set

DNA, Ald-3 = 1-115\3, 369-381\3

DNA, Ald-1 = 2-113\3, 367-382\3

DNA, Ald-2 = 3-114\3, 368-380\3

DNA, Ald-intron = 116-366

DNA, S7-intron = 383-1031

DNA, S7-1 = 1032-1065\3

DNA, S7-2 = 1033-1066\3

DNA, S7-3 = 1034-1067\3

DNA, Tmo-1 = 1068-1626\3

DNA, Tmo-2 = 1069-1624\3

DNA, Tmo-3 = 1070-1625\3

Partitions for the combined mitochondrial data set:

DNA, CR = 1-1084

DNA, 16S = 1085-1606

DNA, 12S = 1607-1952

DNA, ND4-1 = 1954-2653\3

DNA, ND4-2 = 1955-2651\3

DNA, ND4-3 = 1953-2652\3

DNA, ND4-tRNA = 2654-2790

Partitions for the combined data set:

DNA, Ald-3 = 1-115\3, 369-381\3

DNA, Ald-1 = 2-113\3, 367-382\3

DNA, Ald-2 = 3-114\3, 368-380\3

DNA, Ald-intron = 116-366

DNA, S7-intron = 383-1031

DNA, S7-1 = 1032-1065\3

DNA, S7-2 = 1033-1066\3

DNA, S7-3 = 1034-1067\3

DNA, Tmo-1 = 1068-1626\3

DNA, Tmo-2 = 1069-1624\3

DNA, Tmo-3 = 1070-1625\3

DNA, CR = 1627-2710

DNA, 16S = 2711-3232

DNA, 12S = 3233-3578

DNA, ND4-1 = 3580-4270\3

DNA, ND4-2 = 3581-4268\3

DNA, ND4-3 = 3579-4269\3

DNA, tRNA = 4271-4416
