# Supplementary material for: A spectacular new species of seadragon (Syngnathidae)
Source: R Soc Open Sci. 2015 Feb 18;2(2):140458. doi: 10.1098/rsos.140458 (PMC4448810; doi:10.1098/rsos.140458)
Supplement: Table S1-S4. Genetic distances between the seadragon species for mitochondrial data (table S1) and nuclear data (table S2-S4). [file rsos140458supp3.docx]

**ELECTRONIC SUPPLEMENTARY MATERIAL**

# A spectacular new species of seadragon (Syngnathidae)

Josefin Stiller, Nerida G. Wilson, and Greg W. Rouse

**Table S1-S4**. Genetic distances between the seadragon species for mitochondrial data (table S1) and nuclear data (table S2-S4).

**Table S1**.

Pairwise sequence divergence (%) for the 4 mitochondrial markers. Uncorrected distances are shown below the diagonal and GTR+G corrected distances above the diagonal (shape=0.283); diagonal values show intraspecific variability (uncorrected | GTR+G corrected).

|  | *Phycodurus eques* | *Phyllopteryx taeniolatus* | *Phyllopteryx dewysea* n. sp. |
| --- | --- | --- | --- |
| *Phycodurus eques* | 0.3 \| 0.3 | 18.9 | 22.3 |
| *Phyllopteryx taeniolatus* | 11.6 | 1.0 \| 1.1 | 9.9 |
| *Phyllopteryx dewysea* n. sp. | 13.1 | 7.4 | 0.3 \| 0.3 |

**Table** **S2**.

Pairwise sequence divergence (%) for the nuclear ribosomal S7 protein gene. Uncorrected distances are shown below the diagonal and HKY corrected distances above the diagonal; diagonal values show intraspecific variability (uncorrected | HKY corrected).

|  | *Phycodurus eques* | *Phyllopteryx taeniolatus* | *Phyllopteryx dewysea* n. sp. |
| --- | --- | --- | --- |
| *Phycodurus eques* | 0.2 \| 0.2 | 4.1 | 3.5 |
| *Phyllopteryx taeniolatus* | 4.0 | 0.2 \| 0.2 | 2.9 |
| *Phyllopteryx dewysea* n. sp. | 3.4 | 2.9 | 0.0 \| 0.0 |

**Table S3**.

Pairwise sequence divergence (%) for the nuclear Aldolase-like protein gene. Uncorrected distances are shown below the diagonal and HKY corrected distances above the diagonal; diagonal values show intraspecific variability (uncorrected | HKY corrected).

|  | *Phycodurus eques* | *Phyllopteryx taeniolatus* | *Phyllopteryx dewysea* n. sp. |
| --- | --- | --- | --- |
| *Phycodurus eques* | 0.0 \| 0.0 | 2.7 | 2.9 |
| *Phyllopteryx taeniolatus* | 2.6 | 0.0 \| 0.0 | 2.4 |
| *Phyllopteryx dewysea* n. sp. | 2.9 | 2.4 | 0.0 \| 0.0 |

**Table** **S4.**

Pairwise sequence divergence (%) for the nuclear Tmo4c4 protein gene. Uncorrected distances are shown below the diagonal and K80+I corrected distances (pinvar=0.723) above the diagonal; diagonal values show intraspecific variability (uncorrected | K80+I corrected).

|  | *Phycodurus eques* | *Phyllopteryx taeniolatus* | *Phyllopteryx dewysea* n. sp. |
| --- | --- | --- | --- |
| *Phycodurus eques* | 0.0 \| 0.0 | 1.0 | 0.9 |
| *Phyllopteryx taeniolatus* | 1.0 | 0.1 \| 0.1 | 0.8 |
| *Phyllopteryx dewysea* n. sp. | 0.9 | 0.8 | 0.0 \| 0.0 |
